# Popularization of Carbon Capture and Storage Technology in Society: Principles and Methods

**DOI:** 10.3390/ijerph17228368

**Published:** 2020-11-12

**Authors:** Alexey Cherepovitsyn, Tatiana Chvileva, Sergey Fedoseev

**Affiliations:** 1 Organization and Management Department, Saint Petersburg Mining University, 2, 21st Line, 199106 Saint-Petersburg, Russia; Alekseicherepov@inbox.ru; 2 Luzin Institute for Economic Studies—Subdivision of the Federal Research Centre “Kola Science Centre of the Russian Academy of Sciences”, 24a, Fersman St., 184209 Apatity, Russia; S.fedoseev@ksc.ru

**Keywords:** environmental technology, carbon capture and storage, carbon dioxide, popularization of technology, public perception, social license to operate

## Abstract

The problem of global warming is a key challenge. One means to prevent climate change is to reduce the concentration of carbon dioxide in the atmosphere. This can be achieved using CO_2_ capture and storage (CCS) technology. Due to the relative novelty of the technology, low level of experience, and high risk of implementation, in practice society often displays a negative attitude towards CCS projects. Thus, it is necessary to develop a targeted strategy to popularize CO_2_ capture and storage technology. Based on an extensive literature review and the experience of implementation of CCS projects in different countries, this study demonstrates the necessity of applying the deficit, contextual, lay expertise, and public participation models to promote CCS technology. As a result, the factors influencing the choice of promotion tools are identified, and the measures to popularize CCS technology, depending on the stage of its implementation, are determined. Recommendations for the improvement of CCS public databases are developed. The methodologies used this study include case studies, system-oriented analysis, and stakeholder management tools.

## 1. Introduction

Technological development has a significant influence on modern society and has helped address a number of global problems, including climate change, resource depletion, and ecosystem loss [1,2,3,4]. However, in recent decades, the academic community has faced questions about the development of the theoretical aspects of human knowledge and the popularization of the results of scientific studies [5,6,7]. Due to a lack of understanding of new technology and concerns about its safety, the public may not approve the use of some technological solutions in practice. To obtain a social license to introduce new technologies, federal, state, and local governments, the scientific community, and private businesses should develop and implement goal-oriented strategies aimed at receiving approval for their activities from a wide range of stakeholders [8].

In recent decades, the problem of climate change has become one of the most discussed issues internationally. According to the Intergovernmental Panel on Climate Change’s (IPCC) [9], “several regional changes in climate are assessed to occur with global warming up to 1.5 °C compared to pre-industrial levels, including warming of extreme temperatures in many regions (high confidence), increases in frequency, intensity, and/or amount of heavy precipitation in several regions (high confidence), and an increase in intensity or frequency of droughts in some regions (medium confidence)” [9].

On 23 June 1988, Dr. James Hansen (an Adjunct Professor at Columbia University’s Earth Institute (USA), where he directs a program in Climate Science, Awareness and Solutions) testified before the U.S. Senate Committee on Energy and Natural Resources that “the warming trend was not a natural variation but was caused by a buildup of carbon dioxide and other artificial gases in the atmosphere” [10]. Since the late 1970s, James Hansen has focused his research on Earth’s climate and, in particular, anthropogenic climate change. Hansen’s testimony helped to increase public awareness of global warming. Subsequently, a cause and effect relationship between the greenhouse effect and climate change has been confirmed by international academic research [11,12,13,14].

Anthropogenic greenhouse gas emissions are dominated by fossil CO_2_ [15,16]. According to the BP Statistical Review of World Energy 2020 [17], the volume of global carbon dioxide emissions (through consumption of oil, gas, and coal for combustion-related activities) was 34,169 billion tons in 2019, which was 0.5% higher than in 2018 [17]. CO_2_ emissions in 2019 were about 60.1% higher than in 1990 and 44.3% higher than in 2000 [18].

The importance of reducing environmental pollution and carbon dioxide emissions has been emphasized in the studies of numerous researchers [14,19,20,21,22,23]. At the global level, climate change has become an important influencing on the decision-making process of governments, organizations, and societies, particularly in economically developed countries. Strict requirements exist globally to reduce greenhouse gas emissions. For example, the European Union has committed to being climate-neutral by 2050 [24]. In September 2020, the European Commission proposed raising its 2030 greenhouse gas emission reduction target to at least 55% compared to 1990 [25,26]. The existing target for 2030 is to reduce greenhouse gas emissions to at least 40% compared to 1990 [26].

The implementation of new more efficient and environmentally friendly technologies is an important part of low-emission development strategies (LEDS). Carbon capture and storage (CCS) and carbon capture and utilization (CCU) technologies are considered options for reducing anthropogenic carbon dioxide emissions [27,28,29,30]. Carbon capture and storage involves capturing carbon dioxide from emission sources, transporting it to a storage site, and burying it in a suitable underground geological formation [31]. Carbon capture and utilization involves the capture of CO_2_ from emission sources and its subsequent use as a resource to create valuable products or services [23].

Carbon capture and storage technology began to be applied in the 1920s to separate carbon dioxide found in gas fields from commercial methane. In the early 1970s, CO_2_ was captured at a gas processing plant in Texas (USA), transported by pipeline to a nearby oil field, and then injected into reservoirs for enhanced oil recovery [32]. Today the efficiency of CCS and CCU technologies is proven by the successful realization of a large number of projects around the world [30,33].

The technology of sequestration of CO_2_ (for example, carbon farming or urban forestry) also has proven to be an effective measure for the reduction in carbon dioxide in the atmosphere [34,35,36,37,38].

However, in our opinion, CCS, CCU, and sequestration of CO_2_ technologies are characterized by various risks, and, as a result, are perceived differently by a wide range of stakeholders [39,40,41,42]. Thus, in our research we considered carbon capture and storage technology.

CCS projects are accompanied by a large number of risks, including leaks, accidents, environmental pollution, and danger to public health [43,44], and can have significant negative impacts on a wide range of stakeholders, particularly local communities [45,46,47,48,49]. This increases the social risks associated with the implementation of relevant projects.

The relative novelty of carbon capture and storage technology, low level of experience, and high risk of implementation often translate into a negative public perception of CCS in practice. Stakeholders make decisions about their attitude to a project without a clear understanding of all aspects of CCS technology. Separate groups of stakeholders frequently oppose the implementation of carbon capture and storage technology [50]. This can result in the delay or postponement and, in some cases, the cancellation of project activities [51].

Thus, the implementation of measures aimed at the popularization of innovative environmental technologies (including carbon capture and storage) is required. This will help raise awareness of CCS content, risks, and social benefits [52], and will allow stakeholders to make more informed and rational decisions regarding the implementation of specific projects.

The aim of this study was to find and justify the effective ways to promote carbon capture and storage technology to obtain a social license for its implementation.

The key objectives of the paper are:

− Analysis of the literature dedicated to modern approaches to the popularization of new technologies in society;
− Identification of the factors affecting the choice of tools for the popularization of CCS technology in society and the effectiveness of their application;
− Development of the principles of CCS technology popularization;
− Identification of advantages and disadvantages of various methods of popularization of carbon dioxide capture and storage technology.

## 2. Materials and Methods

**Figure 1:**
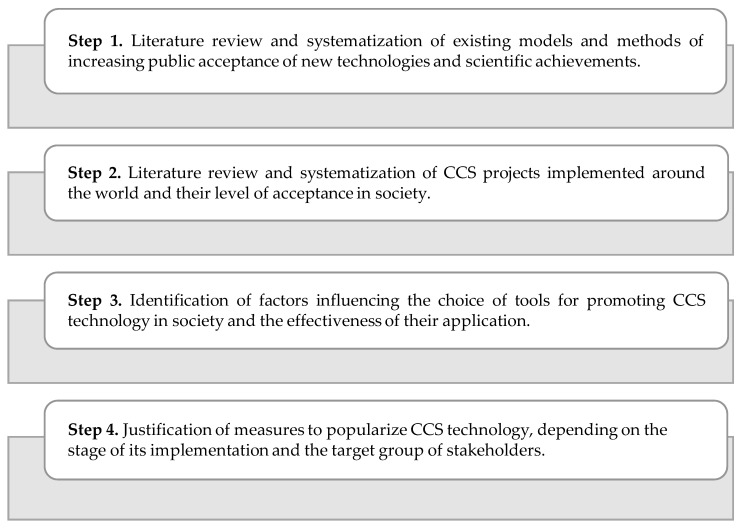
Scheme of the research framework.

The research framework includes four steps (Figure 1).

In each of these individual steps, various methods and materials were used, as summarized below.

Steps 1 and 2: A desk study was carried out to generalize, analyze, and systematize, firstly, information on the assessment of societal attitudes towards the development of science and technology, and the methods of increasing public awareness in this area, and secondly, information on CCS projects implemented around the world and their level of acceptance and approval by stakeholders.

The purpose of step 1 was to investigate modern approaches to the popularization of science and new technologies in society. This step included an overview of the reports developed by the Royal Society (UK) [53], the Lords Science and Technology Committee (UK) [54], and the Research Bureau Limited [55]. The analysis also included a revision of the available reviewed papers published on scientific platforms such as Elsevier Science Direct, Elsevier Scopus, and ResearchGate. The selection of the literature was made based on a few keywords, such as “technology popularization”, “science popularization”, and “science and society”.

The purpose of step 2 was to analyze the implementation progress of CCS projects and identify the key reasons why projects were accepted or rejected by stakeholders. The study included a comprehensive analysis of:

− Open-access databases (the National Energy Technology Laboratory’s Carbon Capture and Storage Database [56], the database of CCS facilities of the Global CCS Institute [57], and the CCS project database provided by the Carbon Capture and Sequestration Technologies at MIT [58]);
− Reports, outlooks, statistics, and data of the Global CCS Institute [59,60], the Intergovernmental Panel on Climate Change (IPCC) [9,61], and the Institute for Sustainable Energy, Environment and Economy [62];
− Available reviewed papers. The analysis was conducted based on a few keywords, such as “CCS project”, “carbon capture and storage”, “social license to operate”, “CCS project perception”, and “CCS project attitude”.

The case studies method was used in this part of the research.

Step 3: The inductive method (deriving conclusions about influences on the effectiveness of the various popularization methods from individual cases of carbon capture and storage projects), synthesis (combining different aspects of public attitude to CCS technology), case studies method, and stakeholder management tools were used in this part of the research.

Step 4: The purpose of step 4 was to adopt existing models for promoting science and its achievements in society, and to develop an approach to carbon capture and storage technology popularization, taking into account its specific features. The case studies method, system-oriented analysis, stakeholder management tools, and project management tools were used in this part of the research.

CCS projects are characterized by multiple and varied dimensions, including social, technological, economic, and environmental dimensions. This paper examines research from three dimensions: social aspects of CCS projects (society’s attitude to CCS technologies, issues of safety for present and future generations, social effect), environmental aspects of CCS projects (proven ability of CCS technologies to reduce emissions of anthropogenic carbon dioxide and influence climate change), and technological aspects of CCS projects (complex technological chain (capture, transportation, injection), accompanied by significant risks, including leaks and accidents).

## 3. Theoretical Framework

Issues related to the attitude of society regarding the development of scientific knowledge, understanding of new technologies, and recognition of their value began to be raised by researchers in the second half of the 20th century. One of the fundamental works in this area is the report of the Royal Society (UK) entitled “The Public Understanding of Science” (1985) [53]. The report recognized a need to monitor and assess society’s attitudes towards the development of science and technology. According to “The Public Understanding of Science”, it is necessary to increase public awareness in this area, including through the implementation of educational programs. Consistent with the recommendations from the Royal Society, the key goal of interaction between scientists, industry, and society is to raise awareness of new technologies that will contribute to their public approval. This concept is called the public understanding of science (PUS) [63,64].

In the 1990s, research was conducted to identify a correlation between the level of knowledge about new technologies and attitudes towards their use. It was found that understanding by stakeholders of the content, risks, and benefits of the implementation of new technologies does not always lead to the formation of a favorable public perception [65,66,67].

Considering the results of the above-mentioned studies, in addition to rapid technological change, the concept of public understanding of science (PUS) was deemed irrelevant. It was replaced by a new concept: “public engagement with science and technology” (PEST) [68,69].

In 2000, the Lords Science and Technology Committee (UK), in their report “Science and Society”, recommended moving from informing society about the results of technological change, to actively involving society in the decision-making process related to the development and implementation of new technologies. This process of involvement must be carried out with the use of public consultation tools, focus groups, participation of stakeholders in panel discussions, and conferences [54].

Within the framework of this approach, various models of popularization of scientific knowledge and new technologies were formulated. Thus, in the report “Science and the Public: Mapping Science Communication Activities” (Research International), three models were suggested: the deficit model, the consultation model, and the engagement model [55]. Bruce V. Lewenstein proposed four models for the popularization of science and its achievements in society: the deficit model, the contextual model, the lay expertise model, and the public participation model [70].

In 2016, the Lords Science and Technology Committee (UK) published “Science communication and engagement”, which noted the importance of implementing a state policy of involving society in the decision-making process regarding scientific and technological development [71].

The evolution of a social license concept has reinforced the importance of actively involving stakeholders in the development and implementation of new technologies [72,73]. This concept involves the active participation of local communities and other stakeholders in the planning and implementation of industry projects [74,75]. Furthermore, consistent with this concept, it is necessary to track the opinions and interests of society [76,77].

In recent years, characterized by the rapid development of Internet technologies, the toolkit for interaction with stakeholders in the framework of practical implementation of achievements of scientific and technological progress has expanded. This, in a report of the National Academies of Sciences (USA) entitled “Communicating Science Effectively: A Research Agenda”, social networks are considered an effective tool for promoting scientific thought and new technologies in society [78].

The considered practices of popularizing scientific achievements and new technologies in society can be applied within the framework of building a strategy for promoting carbon capture and storage technology. However, it is necessary to take into account specific characteristics of CCS.

CCS technology is applicable to different industries (natural gas processing, power generation, iron and steel production, etc.). The key prerequisites for the implementation of CCS projects include the following:

− Availability of permanent sources of CO_2_ emissions;
− Availability of underground storage located close to sources (for example, deposits at a late stage of development and deep-lying aquifers);
− Availability of prospects for creating infrastructure for the implementation of the project.

However, implementation of carbon capture and storage is accompanied by a number of barriers that need to be overcome [79,80]:

1. Technical barriers.

CCS consists of a number of complex processes, including CO_2_ separation, compression, transport, injection into underground reservoirs, and long-term monitoring. The implementation of these processes can be accompanied by leaks, accidents, environmental pollution, danger to public health, etc.

2. Legal and regulatory barriers.

Because of its relative novelty, in a number of countries in which the implementation of CCS technology has significant potential, no specific legislation exists that regulates such projects. Prior to a CCS project’s implementation, it is necessary to introduce clear legislation for CO_2_ capture and storage. A lack of specific legislation causes a CCS project to be postponed or canceled [33].

3. Economic barriers.

CCS projects are characterized by high capital costs, financial problems and risks, and funding problems.

4. Public perception barriers (public awareness and acceptance).

As mentioned previously, separate groups of stakeholders frequently oppose the implementation of carbon capture and storage technology [50].

International experience of the implementation of carbon capture and storage technology shows that public acceptance can be crucial for the success of these projects [50,51]. According to research conducted in Germany, public perception is the second greatest barrier (after economic factors) to the implementation of CCS [81].

Since the 2000s, a significant amount of scientific research devoted to public perception of carbon dioxide capture and storage has been published. The studies can be divided into:

− Publications containing the results of sociological surveys aimed at identifying the attitude of stakeholders to CCS technology and the factors that determine this attitude [62,82,83,84,85];
− Publications reflecting mechanisms of interaction with stakeholders in the framework of carbon dioxide capture and storage projects [59,86,87,88];
− Publications describing experiences of implementation of CCS projects in different countries, the attitude of stakeholders to these projects, and mechanisms of interaction with stakeholders during the projects’ life cycle [51,60,89,90].

Public opinion polls conducted by researchers show a low level of awareness of carbon dioxide capture and storage technology [91] and a high level of concern about its use [92,93]. Thus, despite the experience of implementation of CCS demonstration projects in various countries, including Australia, Japan, and the Netherlands, public perception of this technology is at a relatively low level [94]. This can, in some cases, negatively affect the implementation of specific carbon dioxide capture and storage projects [51].

In spite of the fact that, as mentioned above, the problems of the popularization of science and its achievements are being actively considered by researchers, the most effective methods of promoting CCS technology, taking into account all of its features and risks, have not yet been justified.

## 4. Results

### 4.1. An Approach to the Popularization of Carbon Dioxide Capture and Storage Technology

**Figure 2:**
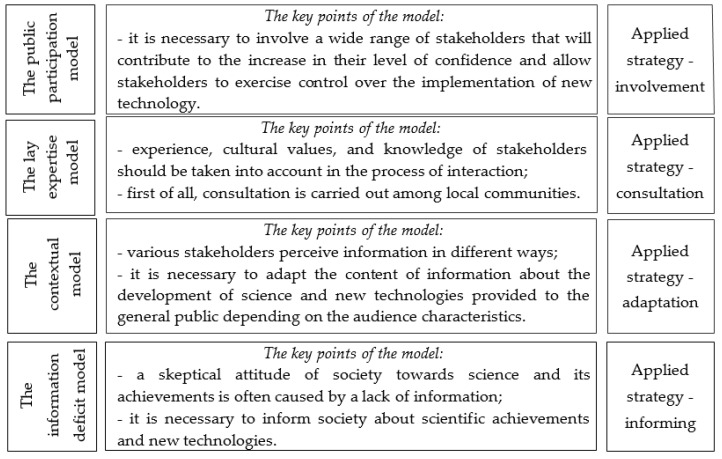
The content of models of popularization of science and technology in society [70]. Compiled by authors based on [70].

As mentioned above, in his work “Models of public communication of science and technology” [70], Bruce V. Lewenstein proposed four models of popularization of science and its achievements in society: the deficit model, the contextual model, the lay expertise model, and the public participation model (Figure 2).

The deficit model is often criticized. Researchers believe that raising awareness of new technologies will not always increase approval of their implementation among a wide range of stakeholders. Thus, the efficiency of the public participation model is recognized [95].

**Figure 3:**
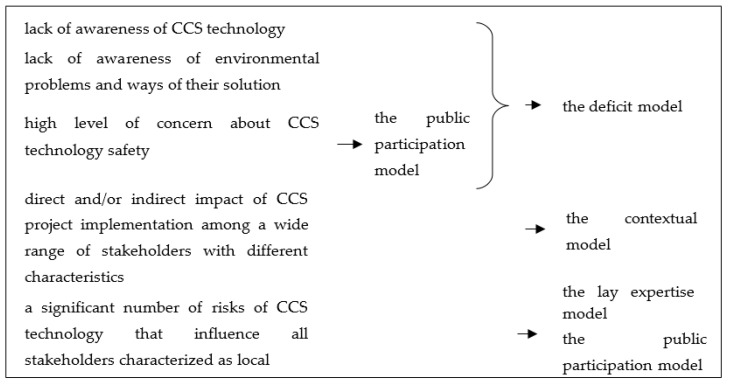
Justification of the application of various models of popularization of carbon capture and storage technology.

Justification of the application of various models of popularization of carbon capture and storage technology is presented in Figure 3.

**Figure 4:**
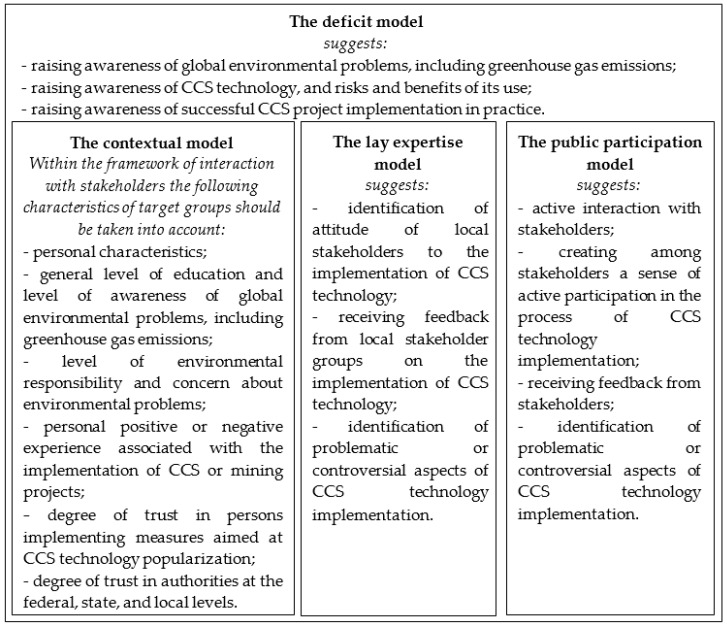
Models of popularization of scientific achievements and new technologies in society in relation to CCS.

The content of models of popularization of scientific achievements and new technologies in society in relation to CCS is presented in Figure 4.

As can be seen in Figure 4, the basic aspect of the popularization of carbon dioxide capture and storage technology is to raise the level of public awareness. The choice of method of interaction with stakeholders should be carried out taking into account the requirements of the contextual model based on the analysis of the following factors:

1. Personal characteristics of the target group, such as age, social status, income level, and gender.

A strategy for the popularization of CCS technology and formation of responsible environmental behavior should encompass people of all ages. The age of the target audience determines the content and specifics of communication.

A number of studies show a higher level of support for environmental technologies among people with higher incomes and social status, compared with representatives of the working class and unemployed [96].

Sociological surveys show a strong correlation between gender and attitudes towards CCS technology. Thus, women are more likely to oppose the use of technology because of doubts about its safety [97]. This determines the development of a strategy for the popularization of CCS projects among women from the perspective of long-term safety [97].

2. General level of education and level of awareness of global environmental problems, including greenhouse gas emissions.

The high level of education determines an increased interest in environmental technologies. In such conditions, a need for information is increasing.

3. Level of environmental responsibility and concern about environmental problems.

The expansion of the concept of sustainable development and rising level of environmental responsibility of civil society indicate an increased interest in environmental technologies and a readiness to perceive information.

4. Personal positive or negative experiences associated with the implementation of CCS or mining projects.

Few people have experience in the implementation of carbon dioxide capture and storage projects. However, related technologies exist that are more prevalent and can also shape the perception of CCS. For example, in a region that is a significant center of oil production, the population has more knowledge and demonstrates a more favorable perception of CCS technology.

5. Regional aspect: the degree of proximity of the target audience to the area of potential or actual CCS project implementation.

The impact of the implementation of CCS projects on various stakeholders is different. The largest number of risks is assumed by local stakeholder groups. This determines the need for continuous interaction with these stakeholders and their active involvement.

6. Degree of trust in persons implementing measures aimed at CCS technology popularization.

The low level of public awareness of carbon dioxide capture and storage technology forces stakeholders to accept the position of experts who are trusted by stakeholders.

7. Degree of trust in authorities at the federal, state, and local levels.

CCS projects require significant government control because of their size and complexity. Civil society will be more favorable to the implementation of carbon dioxide capture and storage projects, knowing that their implementation is monitored and controlled by competent authorities. State participation in the implementation of measures to popularize CCS technology will also contribute to its positive perception.

The choice of a specific method of carbon dioxide capture and storage technology popularization must be made on the basis of a preliminary analysis of the target audience (its boundaries, age, awareness, etc.).

In accordance with Figure 3 and Figure 4, it is necessary to comprehensively apply methods aimed, firstly, at raising public awareness and, secondly, at actively involving stakeholders in the processes of CCS project implementation.

**Figure 5:**
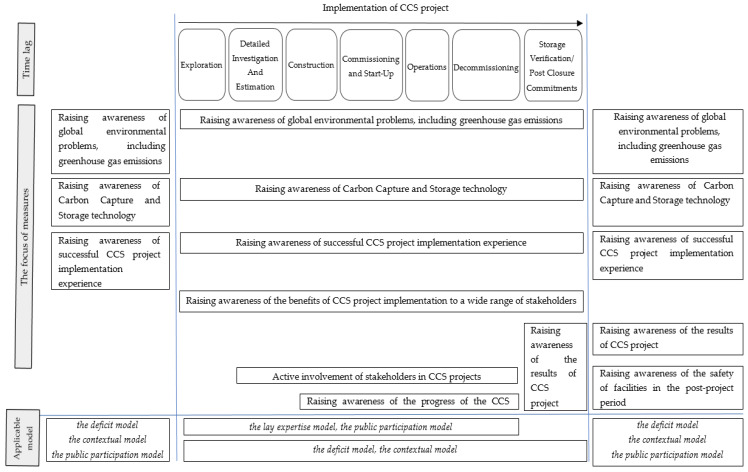
Direction of measures to popularize CCS technology, depending on the stage of its implementation.

Figure 5 shows the focus of measures of CCS technologies’ popularization, depending on the stage of their implementation.

Specific methods that can be used, and their advantages and disadvantages, are presented in Table 1.

To increase their effectiveness, measures aimed at CCS technology popularization should be carried out in compliance with the following principles:

1. Timeliness. Engagement with a wide range of stakeholders should begin before the launch of a CCS project. This allows active involvement of interested groups in discussions of significant aspects of the application of carbon dioxide capture and storage technology, in addition to demonstrating that the opinion of civil society has value.
2. Accessibility of information. The global nature of CCS technology implementation determines the need for free access of a wide range of stakeholders to information.
3. Clarity. It is necessary to make information understandable to stakeholders, taking into account the characteristics of the target group.
4. Balance. The implemented methods of carbon dioxide capture and storage technology popularization should disclose both positive and negative aspects of its application.
5. Monitoring and response. Monitoring allows changes in the public perception of CCS technology to be tracked. This also provides information for making a decision about whether any action to adjust the applied popularization strategy is required.
6. Involvement of independent experts. The low level of public trust in business indicates a need to implement state and regional programs to popularize CCS projects with the involvement of independent experts and representatives of academic institutions.

### 4.2. Recommendations for the Improvement of Public Databases on the World Practice of Carbon Dioxide Capture and Storage Technology Implementation

The methods of promoting carbon dioxide capture and storage technology, described above, will help to increase public awareness of all aspects of CCS. However, an important contribution to its widespread acceptance can be made by providing the interested persons with the information on successful global implementation of carbon dioxide capture and storage projects. This will demonstrate the long-term safety and value of the technology.

Despite the relative novelty of CCS technology, according to the National Energy Technology Laboratory’s (NETL) Carbon Capture and Storage (CCS) Database [56], carbon dioxide capture and storage technology has been implemented in more than 300 projects in more than 30 countries. Nevertheless, about 25 percent of these projects are frozen, more than 20 percent were canceled as a result of management decisions, and about 10 percent were suspended [33]; 93 projects are in an active stage of implementation, 36 are in development.

Thus, significant experience in the implementation of CO_2_ capture and storage projects has been accumulated globally. This can be used to demonstrate to a wide range of stakeholders the practical aspects of the application of CCS technology, including the negative and positive consequences of its implementation.

Several public databases contain information on CCS projects (Table 2)

The analysis of existing databases on CCS projects allows us to draw the following conclusions:

− The databases are public, thus, information is available to any interested person;
− The databases contain general information about CCS project implementation (place of implementation, name, start date, capital intensity, etc.) and information on technical characteristics (type of project, capacity, etc.);
− The databases do not disclose the consequences of risks confirmed during the projects’ implementation, or the impact of these projects on stakeholders;
− Data in the databases are presented in English, which may hinder access to information for non-native English stakeholders;
− The databases contain a list of suspended/closed projects, but the reasons for this are not disclosed.

It should be noted that the CCS project database provided by the Carbon Capture and Sequestration Technologies at MIT [58] contains information on stakeholder perceptions of some projects. However, firstly, this information is random and partial, and secondly, the database was frozen on 30 September 2016.

Thus, we recommend the disclosure of the following significant aspects of the implementation of CCS projects via public databases:

− Information on the reasons for freezing or closing CCS projects;
− Information on the presence/absence of industrial accidents and CO_2_ leaks that occurred during the implementation of CCS projects;
− Information on negative consequences of industrial accidents and CO_2_ leaks;
− Description of the mechanisms for monitoring the progress of CCS projects;
− List of organizations and independent observers exercising control over the progress of CCS project implementation;
− Information on social programs implemented during the project and aimed at local communities, infrastructure development, etc.;
− Information on potential CCS projects.

## 5. Discussion

In our opinion, the development of a strategy for carbon dioxide capture and storage technology popularization needs to be carried out taking into account the following provisions:

1. The public has limited knowledge about CCS technology. This is typical in countries that do not have experience in CO_2_ capture and storage, or in which similar projects have been implemented in the past or are being implemented at present.
2. Modern society expresses a high level of concern about environmental problems. CCS technology is one solution to these problems. Thus, the popularization of CCS projects can be based on raising public awareness of environmental problems and the perception of CO_2_ capture and storage technology as the means to solve them.
3. The public shows a high level of concern regarding the safety of CCS technology due to its relative novelty.
4. Carbon dioxide capture and storage projects are local. However, they are characterized by a high degree of risk, and directly or indirectly affect the interests of a wide range of stakeholders [98]. The degree of impact on various stakeholders is different. Thus, the popularization of carbon dioxide capture and storage technology among stakeholders who cannot be characterized as local (from the perspective of the project area) should be based on raising awareness of modern environmental challenges, the content of CCS technology, and the results of implementation of the demonstration projects in different countries.
5. The largest number of risks, including environmental pollution and health damage, is assumed by local stakeholder groups. Thus, the popularization of carbon dioxide capture and storage technology among stakeholders characterized as local (from the perspective of the project area) should be based on raising awareness of environmental challenges, the content of CCS technology, and the results of implementation of the demonstration projects. Furthermore, an effective popularization strategy should additionally include methods of consultation and active involvement of local stakeholders. Government authorities should also be actively involved in the implementation of carbon dioxide capture and storage projects.

In our opinion, the popularization of CCS technology should be based on four analyzed models (the deficit, contextual, lay expertise, and public participation models).

The practical application of the key research results will contribute to the creation of favorable conditions for the global introduction of carbon dioxide capture and storage technology.

At present, there is a significant amount of research devoted to the application of CCS technology in practice [61,99,100] and the importance of interaction with stakeholders [51,59,60,88]. Although, as mentioned above, the problems of promoting science and its achievements are being actively considered by researchers, in the scientific literature, the most effective methods for the popularization of carbon capture and storage have not yet been determined, taking into account all its features and risks. In addition, CCS technology has significant potential to solve global environmental problems.

Thus, the authors believe that the key difference between this and previous studies is the developed approach to promoting carbon capture and storage in society on the basis of the global experience of the implementation of CCS projects, and its associated features and risks. The recommended improvement of public databases on the global experience of the implementation of carbon dioxide capture and storage technology will increase the access of a wide range of stakeholders to information. This will allow them to make informed decisions about supporting or opposing individual CCS projects.

The main limitations of the paper are the following:

− Open sources of information were used, so some data on CCS projects may be slightly distorted;
− Although the effectiveness of the application of different methods of popularization of carbon capture and storage technology can vary, it was not evaluated. The authors see this as a direction for further research.

## 6. Conclusions

CO_2_ capture and storage projects are characterized by a high degree of risk. Furthermore, they directly or indirectly affect the interests of a wide range of stakeholders. Thus, to reduce the social risks of technology implementation and increase the efficiency of specific projects, it is necessary to obtain a social license from society.

To develop the most effective strategy for promoting CCS technology, it is necessary to carry out preparatory work aimed at assessing the target audience, and identifying the audience’s level of awareness and the reasons for positive or negative attitudes towards technology. The strategy should include a range of measures aimed at raising public awareness of the technology, and its risks and benefits, and actively involving stakeholders in the process of CCS implementation.

The strategy of popularization of carbon dioxide capture and storage technology among stakeholders who cannot be characterized as local should include educational methods and media coverage. The aim should be to raise awareness of modern environmental challenges; the content, risks, and benefits of CCS technology; and the results of implementation of demonstration projects in different countries.

The popularization of carbon dioxide capture and storage technology among local stakeholders, in addition to educational methods and media coverage, should also include methods of consultation and active involvement, such as consensus conferences, stakeholder meetings, visits to a project site, and meetings at information centers.

One of the key components of the popularization of carbon dioxide capture and storage technology is the availability and reliability of related information and the results of the implementation of specific projects for a wide range of stakeholders.

## Figures and Tables

**Table 1:** Methods of popularization of carbon dioxide capture and storage technology and CCS projects.

Method	Objectives of the Method	Target Group	Advantages	Disadvantages
*Educational*				
Courses dedicated to the environmental challenges facing modern society, the development of technologies aimed at their solutions, implemented within the educational programs of secondary and higher education.	Informing	Students	− Allows creation among young people of ideas about ecological problems and environmental protection technologies, and responsible behavior; − Allows coverage of a wide range of issues; − There is a dialogue that contributes to better understanding of information.	− A limited audience.
Courses distributed by online platforms devoted to the environmental challenges facing modern society, the development of technologies aimed at their solution, and also to disclosure of the content, risks, and benefits of CCS technology.	Informing	General public with access to the Internet	− Allows a large audience to be reached; − Makes it possible to visualize technology; − Allows coverage of a wide range of issues; − There is a dialogue that contributes to better understanding of information; − Provides easy access to information; − Enables permanent access to information.	− Courses are usually limited in time; − Method does not cover an audience that does not have access to a computer and the Internet.
Educational programs (professional development programs, retraining of personnel) dedicated to the environmental challenges facing modern society, the development of technologies aimed at their solution, and also to disclosure of the content, risks, and benefits of CCS technology.	Informing	Representatives of organizations operating in the field of environmental protection, oil production, etc.	− Those who enroll in the course are usually motivated to study; − Raises awareness of environmental protection technologies among those who can influence decisionmaking on CCS implementation; − Makes it possible to study in depth a wide range of issues; − There is a dialogue that contributes to better understanding of information.	− A limited audience; − Programs are usually limited in time.
*Media*				
Videos or popular science movies and TV shows devoted to environmental problems and environmental technologies, including CCS technology.	Informing	General public	− Can be broadcast on TV channels, reaching the widest possible audience; − Can be used repeatedly during presentations, meetings, etc.; − Makes it possible to visualize technology.	− Videos, popular science movies, and TV shows that touch on complex, technical issues may not be fully understood by the audience; − High financial costs; − Method does not allow the audience to ask questions, there is no dialogue.
Coverage of environmental problems and environmental technologies, including CCS technology, in the print media.	Informing	General public	− Provides the widest possible audience coverage; − Makes it possible to visualize technology.	− Complex, technical issues may not be fully understood by the audience; − High financial costs; − Method does not allow the audience to ask questions, there is no dialogue.
Websites devoted to carbon capture and storage technology and specific CCS projects.	Informing/ involvement	General public with access to the Internet	− Provides easy access to information; − Enables permanent access to information; − It is possible to update information; − Allows links to independent resources to be added; − May include online forums where experts and stakeholders can discuss CCS technology or specific projects; − Makes it possible to cover a wide range of issues; − Allows a large audience to be reached; − Can be used to collect feedback.	− Method does not cover an audience that does not have access to a computer and the Internet.
Coverage of environmental issues and environmental technologies, including CCS technology, in social networks	Informing/ involvement	General public with access to the Internet	− Provides easy access to information; − Enables permanent access to information; − It is possible to update information; − Allows links to independent resources to be added; − Makes it possible to cover a wide range of issues; − Allows a large audience to be reached; − Can be used to collect feedback.	− Method does not cover an audience that does not have access to a computer and the Internet.
Distribution of printed materials (flyers, brochures, posters, etc.)	Informing	General public	− Allows a large audience to be reached; − Makes it possible to cover a wide range of issues; − Makes it possible to visualize technology.	− Stakeholders often do not read printed materials; − Method does not allow the audience to ask questions, there is no dialogue.
*Event*				
Lectures and presentations on environmental issues and environmental technologies, including CCS technology	Informing	General public	− Participation of specialists improves the quality and content of the information provided; − Can be used to collect feedback; − There is a dialogue that contributes to better understanding of information; − It is possible to invite media to cover events.	− A limited audience; − Lectures and presentations are limited in time; − Complex, technical issues may not be fully understood by the audience.
Consensus conference	Informing/ involvement	General public, local communities	− Allows involvement of stakeholders and identification of their attitude to CCS technology; − Participation of specialists improves the quality and content of the information provided; − Can be used to collect feedback; − There is a dialogue that contributes to better understanding of information; − It is possible to invite media to cover events.	− The views of participants of the consensus conference may not reflect the views of the general public; − A limited audience; − Consensus conferences are usually limited in time.
Exhibitions devoted to environmental problems and environmental technologies, including CCS technology	Informing	General public	− Participation of specialists improves the quality and content of the information provided; − There is a dialogue that contributes to better understanding of information; − It is possible to invite media to cover events.	− A limited audience; − Usually limited in time; − Complex, technical issues may not be fully understood by the audience.
Information Centers	Informing/ involvement	Local communities	− Enables permanent access to information; − Space can be used for events, including meetings with stakeholders; − Enables stakeholders to receive comments on the implementation of CCS technology.	− A limited audience; − High financial costs.
Stakeholder meetings	Informing/ involvement	General public, local communities	− Allows a full presentation of CCS technology; − Can be used to collect feedback; − There is a dialogue that contributes to better understanding of information; − It is possible to invite media to cover events.	− It can be difficult to discuss all issues that concern stakeholders who attend a meeting; − A limited audience.
Visit a project site	Informing/ involvement	Local communities	− Allows a full presentation of CCS technology; − Can be used to collect feedback; − There is a dialogue that contributes to better understanding of information; − It is possible to invite media to cover events.	− A small number of people can visit the project site; − The need to comply with strict safety rules.

**Table 2:** Existing public databases containing information on the global experience of introducing carbon dioxide capture and storage technology.

Database Name	Information Contained in the Database
The National Energy Technology Laboratory’s (NETL) Carbon Capture and Storage (CCS) Database	Project name, company, plant name, type of project, project overall status, plant status, project phase, country location, state location, specific site location, plant size or capture amount, combustion/separation, capture technology, amount of CO_2_ captured/stored, project summary, project start date, project cost, project information webpage.
CO2RE (a database of CCS facilities of the Global CCS Institute)	Project name, country location, type of project, project overall status, project short description, project start date. CO_2_ emissions data, policy, regulatory and storage emissions data.
The CCS project database provided by the Carbon Capture and Sequestration Technologies at MIT	Project name, company, location, type of capture technology, project overall status, project start date, project cost, economic indicators, project information webpage, project information and comments, including public attitude.

Compiled by authors based on [56,57,58].
